# Deep-proteome mapping of WM-266-4 human metastatic melanoma cells: From oncogenic addiction to druggable targets

**DOI:** 10.1371/journal.pone.0171512

**Published:** 2017-02-03

**Authors:** Eumorphia G. Konstantakou, Athanassios D. Velentzas, Athanasios K. Anagnostopoulos, Zoi I. Litou, Ourania A. Konstandi, Aikaterini F. Giannopoulou, Ema Anastasiadou, Gerassimos E. Voutsinas, George Th. Tsangaris, Dimitrios J. Stravopodis

**Affiliations:** 1 Section of Cell Biology and Biophysics, Department of Biology, School of Science, National and Kapodistrian University of Athens, Athens, Greece; 2 Proteomics Core Facility, Systems Biology Center, Biomedical Research Foundation of the Academy of Athens, Athens, Greece; 3 Basic Research Center, Biomedical Research Foundation of the Academy of Athens, Athens, Greece; 4 Laboratory of Environmental Mutagenesis and Carcinogenesis, Institute of Biosciences and Applications, National Center for Scientific Research “Demokritos”, Athens, Greece; University of Alabama at Birmingham, UNITED STATES

## Abstract

Cutaneous melanoma is a malignant tumor of skin melanocytes that are pigment-producing cells located in the basal layer (stratum basale) of epidermis. Accumulation of genetic mutations within their oncogenes or tumor-suppressor genes compels melanocytes to aberrant proliferation and spread to distant organs of the body, thereby resulting in severe and/or lethal malignancy. Metastatic melanoma’s heavy mutational load, molecular heterogeneity and resistance to therapy necessitate the development of novel biomarkers and drug-based protocols that target key proteins involved in perpetuation of the disease. To this direction, we have herein employed a nano liquid chromatography-tandem mass spectrometry (nLC-MS/MS) proteomics technology to profile the deep-proteome landscape of WM-266-4 human metastatic melanoma cells. Our advanced melanoma-specific catalogue proved to contain 6,681 unique proteins, which likely constitute the hitherto largest single cell-line-derived proteomic collection of the disease. Through engagement of UNIPROT, DAVID, KEGG, PANTHER, INTACT, CYTOSCAPE, dbEMT and GAD bioinformatics resources, WM-266-4 melanoma proteins were categorized according to their sub-cellular compartmentalization, function and tumorigenicity, and successfully reassembled in molecular networks and interactomes. The obtained data dictate the presence of plastically inter-converted sub-populations of non-cancer and cancer stem cells, and also indicate the oncoproteomic resemblance of melanoma to glioma and lung cancer. Intriguingly, WM-266-4 cells seem to be subjected to both epithelial-to-mesenchymal (EMT) and mesenchymal-to-epithelial (MET) programs, with 1433G and ADT3 proteins being identified in the EMT/MET molecular interface. Oncogenic addiction of WM-266-4 cells to autocrine/paracrine signaling of IL17-, DLL3-, FGF(2/13)- and OSTP-dependent sub-routines suggests their critical contribution to the metastatic melanoma chemotherapeutic refractoriness. Interestingly, the 1433G family member that is shared between the BRAF- and EMT/MET-specific interactomes likely emerges as a novel and promising druggable target for the malignancy. Derailed proliferation and metastatic capacity of WM-266-4 cells could also derive from their metabolic addiction to pathways associated with glutamate/ammonia, propanoate and sulfur homeostasis, whose successful targeting may prove beneficial for advanced melanoma-affected patients.

## Introduction

Skin cancer is ranked in the third position among all human malignancies. Its global incidence is rising at alarming levels with 2–3 million cases being estimated to develop each year worldwide [1]. Cutaneous melanoma belongs to the most aggressive and treatment-resistant human cancers, surpassing the majority of solid tumors in terms of their propensity to metastasize [2–4]. In USA, the risk of invasive melanoma has increased almost 10 times during the last 50 years, while in Queensland Australia the cumulative melanoma incidence in citizens of the general population over 50 years old is estimated 1 in 19 for men and 1 in 25 for women [3, 5]. Due to depletion of the stratospheric ozone layer, exposure patterns of earth’s surface to ultraviolet radiation (UVR) become gradually unsafe, prompting the epidemiological and mechanistic association between melanomagenesis and UVR [1–3]. To this direction, UVR has proved capable to accelerate BRAF^V600E^-driven melanomagenesis via induction of critical mutations in the *TP53/p53* genetic locus [6].

Cutaneous melanoma arises from malignant transformation of melanocytes, the melanin-producing cells that control pigmentation and photo-protection of the skin [7]. Dysfunctional signaling in melanocytes disengages them from keratinocytic regulation, and propels their aberrant proliferation and spreading leading to formation of naevi and -common- moles. Even though naevi are generally benign, they can progress through a radial-growth phase to a vertical-growth phase in which cells acquire growth-factor independence and present metastatic potential [1, 3, 8]. Interestingly, the SOX10 transcription factor which is required for melanocyte development is prominently expressed in giant congenital naevi and melanoma, while *SOX10* gene silencing effectively blocks human melanomagenesis *in vivo* [9].

High mutational load and molecular heterogeneity represent major challenges in the comprehensive analysis of melanoma genomes. Next-generation sequencing has unearthed that melanoma is classified among the cancer types with the highest rates of somatic-base mutations [1, 8]. A catalogue of 33,345 somatic-base substitutions was identified in COLO-829 melanoma cells, and it proved to contain mutational signatures of UVR-induced DNA damage and driver mutations that conferred selective growth advantage on tumor cells [10]. The mutational landscape of melanoma has been thoroughly examined, and driver mutations in the *BRAF*, *NRAS*, *KIT*, *GNAQ*, *GNA11*, *NF1*, *CDKN2A*, *PTEN* and *MITF* genetic loci have been recurrently recognized [1–4, 8, 11, 12], with *BRAF* alterations typifying up to ~70% of melanoma tumors [3, 12, 13].

Given its principal role in melanoma initiation and progression, oncogenic BRAF has become a druggable kinase and a crucial target for small-molecule inhibitors. For example, vemurafenib is the first drug having been approved for the treatment of patients diagnosed with *BRAF*^*V600E*^-positive melanomas [4, 8, 11, 12, 14, 15]. However, the acquired resistance to the drug previously reported to emerge from PDGFRβ upregulation [15, 16], NRAS mutations [15, 16], HGF-dependent signaling-network activation [15, 17, 18] and aberrantly spliced BRAF^V600E^ dimerization [15, 19] compromises the successful management of the disease, therefore necessitating the discovery of new regimens with high therapeutic efficiency and low systemic toxicity.

To cope with the cellular and molecular heterogeneity of melanomas, and to identify novel protein biomarkers that may serve as drug targets for the disease, we herein employed a high-resolution liquid chromatography-tandem mass spectrometry (LC-MS/MS) proteomics technology joined with versatile bioinformatics platforms to capture the proteomic landscape of WM-266-4 human metastatic melanoma cells. A comprehensive and likely the hitherto largest single cell-line-derived protein catalogue for the disease (6,681 unique members) was identified, and its contents were categorized according to their cellular topology, function and tumorigenicity. Molecular reconstruction of diverse networks dictated the oncogenic addiction of metastatic melanoma to critical EMT/MET, signaling and metabolic sub-routines, and implied the beneficial value of their targeted drugging to disease-affected patients.

## Materials and methods

### Melanoma cell line and culture conditions

The WM-266-4 cell line, a hypertriploid line of an epithelial and adherent character, was derived from a metastatic site (on the skin) of a malignant melanoma female patient of 58 years old (https://www.lgcstandards-atcc.org/en/Global/Products/2/0/6/B/CRL-1676.aspx) and it was purchased from ECACC/Sigma-Aldrich (Munich, Germany). Cells were obtained from the provider at passage three and further cultured, without detectable morphological changes for all successive passages, in DMEM (supplemented with 10% FBS, 2 mM L-glutamine, 1 mM sodium pyruvate, 50 mM sodium bicarbonate, 1x non-essential amino acids, 100 U/ml penicillin and 100 μg/ml streptomycin) at 37°C and 5% CO_2_. Massive cultures of WM-266-4 cells at their tenth passage were harvested via application of a scraping-based protocol and after three washes with 0.9% NaCl cells were centrifuged at 750 *g* for 10 minutes, and the generated pellet was stored at -20°C for further processing. All culture media and reagents were provided by Merck Millipore-Biochrom AG (Merck KGaA, Darmstadt, Germany).

### WM-266-4-specific xenografts in SCID mice

NOD.CB17-Prkdcscid/J (SCID) immunodeficient mice (The Jackson Laboratory, Maine, USA) were used for metastatic-melanoma xenografts establishment. SCID mice were subcutaneously inoculated with ~5 x 10^5^ WM-266-4 cells per animal and closely followed till the development of tumors. Tumors were excised and photographed thirty days after WM-266-4 cell injections. Experiments were repeated two independent times using different cell cultures and animal colonies. Animals were treated according to Greek laws (2015/92), guidelines of European Union and European Council (86/609 and ETS123, respectively), and in compliance with standards for human care and use of laboratory animals (NIH, USA, assurance no. A5736-01).

### Extraction of proteins and preparation of tryptic peptides

Sample preparation was basically carried out as previously described [20]. Briefly, cell pellets containing ~10^7^ cells were suspended in lysis buffer [1.5 M Tris-HCl (pH 7.6), 3.5 M urea, 0.1 M SDS and 3.2 mM DTE (dithioerythreitol)] and disrupted by tip-sonication. Following lysis, samples were centrifuged at 13,000 *g* for 20 minutes to remove cellular debris and insoluble material. Total protein concentration was measured on supernatants using the Bradford assay. 150 μg of whole-cell lysate from each sample were reduced and alkylated by incubation with 0.1 mM DTE in Tris-HCl (pH 6.8) for 30 minutes at 56°C. Then, proteins were alkylated by addition of 0.05 mM iodoacetamide for 30 minutes at room temperature in the dark. Each sample was treated by trypsin (Roche, Hoffman-La-Roche, Basel, Switzerland) at a protein to trypsin ratio 100:1. Trypsinization was terminated by addition of 5% acetic acid. Peptide-containing solutions were vacuum-dried for 60 minutes and the produced powder was re-constituted in 100 μl of buffer A (0.1% formic acid in ddH_2_O) for LC-MS/MS analysis.

### Nano LC-MS/MS analysis

Nano liquid chromatography-tandem mass spectrometry (nLC-MS/MS) was performed as previously described [20]. Briefly, the peptide mixture from tryptic melanoma-cell digest was separated with a linear gradient of 2–30% buffer B (99.9% acetonitrile and 0.1% formic acid) at a flow rate of 300 nl per minute on a C-18 column (75 μm x 50 cm; 100 Å, 2-μm-bead-packed Acclaim PepMap RSLC; Thermo Fisher Scientific, Rockford, Illinois, USA) in buffer A (0.1% formic acid in ddH_2_O). An Ultimate-3000 system (Dionex, Thermo Scientific, Bremen, Germany) was on-line coupled to an Orbitrap Elite instrument (Thermo Fisher Scientific). Mass spectra were collected in a data-dependent acquisition mode using the XCalibur^™^ v.2.2 SP1.48 software (Thermo Fisher Scientific). Full-scan data were acquired on the 300–2,000 m/z range with resolution set to a value of 60,000 with a maximum injection time of 100 milliseconds. Data-dependent MS/MS for the 20 most intense ions per survey scan was performed with HCD (higher-energy collision dissociation) fragmentation on the Orbitrap at a resolving power of 15,000 and collision energy of 36 NSE%. Produced fragments were analyzed on the Orbitrap, and MS/MS spectra were acquired with 15,000 resolving power and maximum injection time of 120 milliseconds. Measurements were performed using m/z 445.120025 as lock mass, while dynamic exclusion was employed within 45 seconds to prevent repetitive selection of the same peptide.

### Data analysis

Raw data were processed through Proteome Discoverer (Thermo Fisher Scientific), while for protein identification analysis the database employed was the *Homo sapiens* proteome of reference from UNIPROT using Sequest-HT v.28.0 (Thermo Fisher Scientific). The search parameters chosen were: (a) two maximum missed cleavages for trypsin, (b) oxidation of methionine as variable modification, (c) 10 ppm peptide mass tolerance and (d) 0.05 ppm fragment ion tolerance. PSMs (peptide spectral matches) were validated using percolator based on q-values at 1% FDR (false discovery rate). The minimum length of acceptable identified peptides was set as 6 amino acid residues.

### Bioinformatics platforms

Protein-accession numbers as they were retrieved from UNIPROT Knowledgebase v.2.16 [21] (http://www.uniprot.org) were further processed via employment of: (a) DAVID (Database for Annotation, Visualization and Integrated Discovery) 6.7 [22, 23] (http://david.abcc.ncifcrf.gov/home.jsp), (b) KEGG (Kyoto Encyclopedia of Genes and Genomes) [24, 25] (http://www.genome.jp/kegg), (c) PANTHER (Protein ANalysis THrough Evolutionary Relationships) [26, 27] (http://pantherdb.org), (d) INTACT [28] (http://www.ebi.ac.uk/intact), (e) CYTOSCAPE [29] (http://www.cytoscape.org) and (f) dbEMT (Epithelial-Mesenchymal Transition gene database) [30] (http://dbemt.bioinfo-minzhao.org) bioinformatics resources.

## Results and discussion

### Clustering of WM-266-4 melanoma proteins according to their cellular compartmentalization, biological function and tissue-specific oncogenicity

For each one of the two experiments performed (with cell pellets having been derived from independent cell cultures), tryptic peptides of WM-266-4 protein extracts were processed through nano LC-MS/MS analysis on an Orbitrap Elite instrument. UNIPROT employment allowed the identification of 6,681 proteins directly derived from 23,191 unique peptides (S1 Table). This protein collection comes from the cumulative unification of two different deep-proteome catalogues herein produced (each LC-MS/MS analysis has been performed three times). It further expands the WM-266-4 proteomic profiling we have recently reported [20] and likely represents the hitherto largest single cell-line-derived proteomic assembly of the disease.

**Fig 1:**
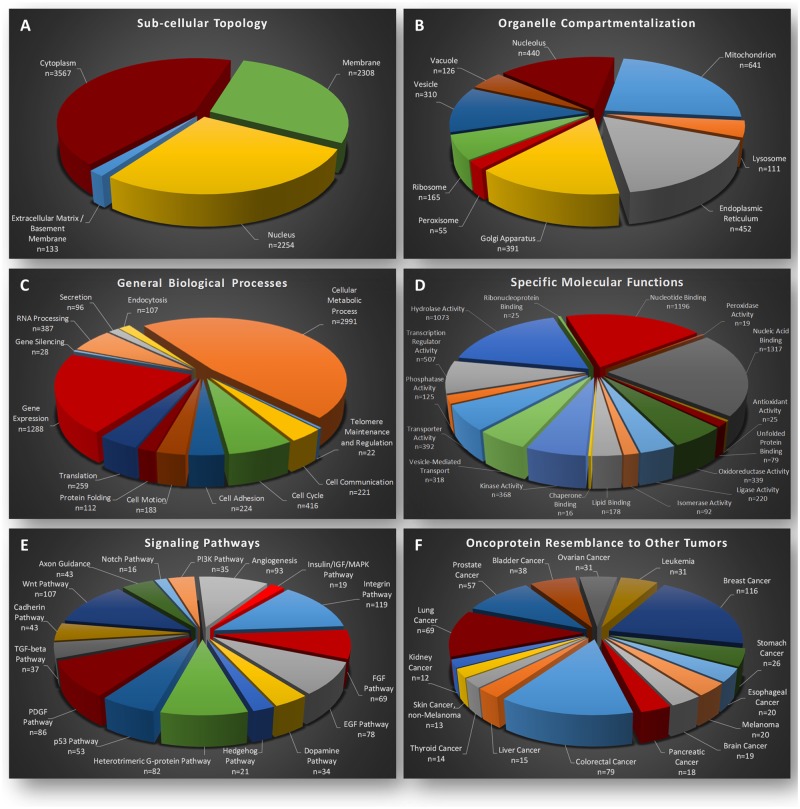
Classification of WM-266-4 deep-proteome components according to their: (A) “sub-cellular topology”, (B) “organelle compartmentalization”, (C) “general biological processes”, (D) “specific molecular functions”, (E) “signaling pathways” and (F) “oncoprotein resemblance to other tumors”. Due to their specific features, certain proteins can be categorized in more than one group. The bioinformatics sub-routines employed were: the (A and B) GO (Gene Ontology) “Cellular Components”, (C) GO “Biological Processes”, (D) GO “Molecular Functions”, (E) PANTHER pathways and (F) GAD. (A-D, F) analyzed through DAVID.

According to the DAVID sub-routine GO (Gene Ontology; “Cellular Components”), the majority of melanoma proteins are classified in the “cytoplasm” (n = 3,567), while both “nucleus” and “membrane” categories prove to be surprisingly enriched (n = 2,254 and n = 2,308, respectively) (Fig 1A), despite the technical limitations of current proteomics protocols to successfully extract nuclear and membrane proteins [31, 32]. Regarding “organelle compartmentalization” (DAVID—GO; “Cellular Components”), most proteins are sorted to “mitochondrion” (n = 641), indicating the high bioenergetic demands of melanoma cells. To the same direction, the intense protein synthesis (“nucleolus” and “ribosome”), trafficking (“vesicle”), stability (“lysosome”) and secretion (“endoplasmic reticulum” and “Golgi apparatus”) are reflected in the large number of components categorized for each respective organelle (Fig 1B).

Approximately 45% (n = 2,991) of all WM-266-4 proteins are presented with “cellular metabolic process” (DAVID—GO; “Biological Processes”) features, suggesting the enhanced bioenergetic requirements of metastatic melanoma cells for survival and growth (Fig 1C). “Gene expression” represents the second category in terms of protein number (n = 1,288; ~19%), indicating the importance of multiple and synergistically orchestrated gene activities in melanoma initiation and progression (Fig 1C). In accordance, 1,317 melanoma proteins carry “nucleic acid binding” (DAVID—GO; “Molecular Functions”) properties, while 507 ones are implicated in “transcription regulator activity” (Fig 1D). The power of melanoma cells to derail their healthy micro-environment and be successfully adapted to new settings during metastasis could be associated with the enriched “transporter activity” (n = 392) and “vesicle-mediated transport” (n = 318) groups (Fig 1D). From the census of WM-266-4 deep kinome (n = 368; “kinase activity”) and phosphatome (n = 125; “phosphatase activity”) components (Fig 1D), multiple kinase and phosphatase cancer drivers may be identified and further exploited as drug targets for the elimination of aberrant signaling in metastatic melanoma cells.

Among the “signaling pathways” examined via the PANTHER bioinformatics sub-routine, “integrin” (n = 119), “Wnt” (n = 107), “angiogenesis” (n = 93), “PDGF” (n = 86), “heterotrimeric G-protein” (n = 82), “EGF” (n = 78) and “FGF” (n = 69) are presented as the largest in content categories (Fig 1E), implying their essential and frequently aberrant roles in melanoma uncontrolled cell cycle, apoptosis resistance, growth-factor independence, angiogenesis, bioenergetic reprogramming and metastasis. It may be one or more of these pathways that likely operate irregularly to promote besides melanoma a number of different human malignancies. Interestingly, by engaging GAD (Genetic Association Database) [33] through the DAVID bioinformatics tool, “breast” (n = 116), “colorectal” (n = 79), “lung” (n = 69) and “prostate” (n = 57) cancer types seem to share multiple oncoproteins with WM-266-4 metastatic melanoma cells (Fig 1F). This supports the notion that a number of common oncoregulators can compel diverse -healthy- tissues to malignancy and provides a rationale for the ability of melanoma cells to metastasize to other organs.

### Melanoma cells acquire tumorigenic plasticity via combined operation of networks controlling melanogenesis and stem-cell pluripotency

**Fig 2:**
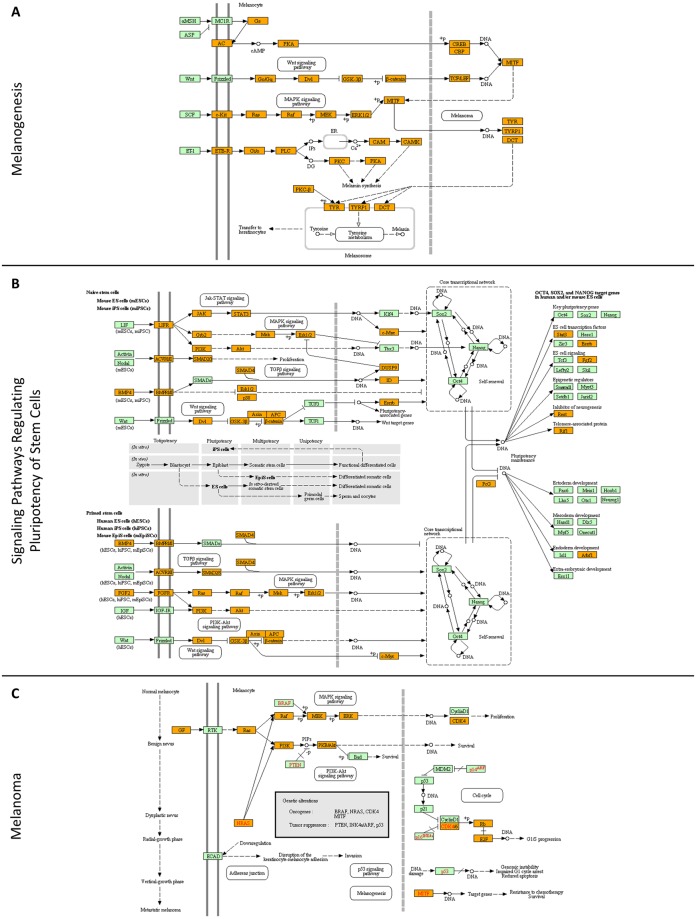
Reconstruction of molecular networks that control: (A) “melanogenesis”, (B) “pluripotency of stem cells” and (C) “melanoma”-formation processes. Orange boxes: WM-266-4 melanoma proteins identified in the present study. Green boxes: proteins that have been missed in this study (for either technical or biological reasons). Red fonts indicate protein products of oncogenes or tumor suppressor genes involved in the disease. The bioinformatics tool engaged was the KEGG pathway maps.

**Fig 3:**
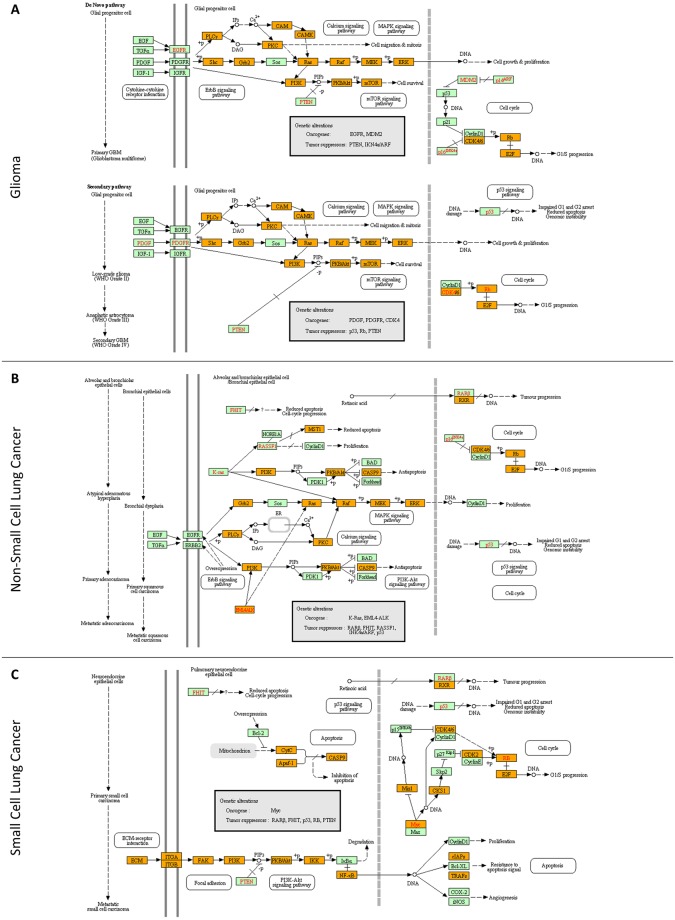
Molecular reassembly of networks that regulate: (A) “glioma”, (B) “non-small cell lung cancer” and (C) “small cell lung cancer” initiation and progression. Orange boxes: WM-266-4 melanoma proteins that have been identified in the present study. Green boxes: proteins that have been missed in this study (for either technical or biological reasons). Red fonts indicate protein products of oncogenes or tumor suppressor genes involved in each disease. The bioinformatics sub-routine employed was the KEGG pathway maps.

Through application of the KEGG bioinformatics software, the molecular networks of “melanogenesis” and “stem-cell pluripotency” could be successfully reconstructed (Fig 2). Interestingly, the identification of MITF (7.64), a master regulator of melanocyte development [34], together with the products of its downstream target genes *TYR* (47.22), *TYRP1* (0.00) and *DCT/TYRP2* (31.41) (number in parenthesis denotes the “Mascot score”) [34–36] suggests that the WM-266-4 cells have maintained (even partly) their melanocytic character (Fig 2A). Nevertheless, they are also presented with autocrine/paracrine signaling of BMP- and FGF-dependent pathways that critically regulate cellular stemness and pluripotency (Fig 2B). Moreover, the identification of SOX5, SOX10, c-MYC, TFAP2A, TFAP2C, RXRG, EBF1 and ID4, critical regulators of neural crest (a multipotent cell population) specification and migration [37–39], dictates the neural-crest developmental origin of WM-266-4 cells. However, CD271, a neural-crest nerve growth factor receptor that is associated with human melanoma tumor stem cells [40], could not be detected in our WM-266-4 deep-proteome catalogue, likely due to technical limitations of proteomics technology. For example, the low abundance of transcription factors and their tight association to nuclear chromatin, together with the reduced solubility of transmembrane proteins may critically compromise LC-MS/MS efficiency [31, 32]. Engaging “Mascot score” as a reliable parameter for protein quantification [41], the SOX10 and MITF transcription factors which are essentially implicated in melanoma formation and maintenance [1, 3, 4, 9, 11, 34, 42] seem to be significantly expressed in WM-266-4 cells. Intriguingly, SOX9, a critical regulator of SOX10 and MITF protein expression [43], was missing from our proteomic collection. It seems that absence of SOX9 renders melanoma cells resistant to T cell-mediated killing, thereby promoting their escape from immune surveillance [44]. This melanoma oncoproteome (Fig 2C) bears notable resemblance to “glioma” (Fig 3A), “non-small cell lung cancer” (Fig 3B) and “small cell lung cancer” (Fig 3C) respective oncoproteomes, likely reflecting the common embryonic origin of melanocytes and glia [34], and also justifying the aggressive potency of melanoma to metastasize to brain and lung [45].

**Fig 4:**
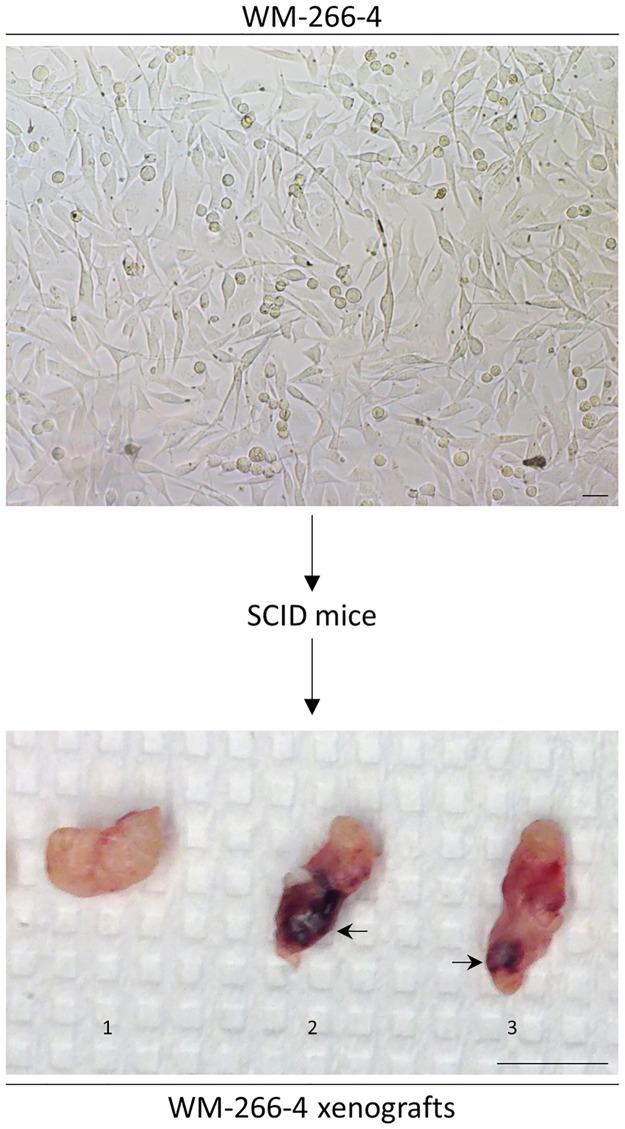
A characteristic image of WM-266-4 metastatic melanoma cells grown *in vitro* (upper panel). WM-266-4 representative melanoma tumor xenografts dissected from SCID mice revealing the heterogeneity of tumors’ (1–3) melanin content (lower panel). 1: melanin-free tumor, 2: melanin-enriched tumor (arrow) and 3: melanin-low tumor (arrow). Scale bars: 50 μm (upper panel) and 1 cm (lower panel).

In order to examine their oncogenic capacity, WM-266-4 cells where grown *in vitro* (as a morphologically rather homogeneous culture, see Fig 4; upper panel) and subsequently injected in SCID mice to produce melanoma tumors in animals’ thighs (Fig 4; lower panel). The WM-266-4-derived xenograft melanoma tumors proved able not only to grow relatively quickly in significant size and weight, but also to exhibit a level of heterogeneity regarding their obtained melanin content. It seems that WM-266-4 cells can generate *in vivo* melanoma tumor xenografts with a melanin production range from none to high (Fig 4; lower panel). This indicates that WM-266-4 cells consist of, at least, two distinct sub-populations that differ from each other in their melanocytic capacity, and moreover suggests that the melanin-positive (or committed to melanin production, under certain settings) (minor; TYRP1: 0.00) sub-population of WM-266-4 cells carries an oncogenic capacity and most likely does not represent a group of melanocytes that was accidentally co-dissected during the process of WM-266-4 initial establishment. Given the previously reported role of melanin in the compromise of radio-, chemo- and immunotherapy efficacy in metastatic melanoma [46–49], it could be the melanin-positive sub-populations, like the one observed in WM-266-4 cells, that confer diverse types of resistance frequently developed during therapy. Enhanced synthesis of melanin implies increased intracellular concentration of L-tyrosine [7, 50] and therefore upregulated levels of fumarate, a metabolic intermediate generated, among others, by the decomposition process of L-tyrosine. Thus, we herein suggest that the melanin-positive sub-population carries advanced fumarate levels, and based on a recently published work in renal cell cancer [51], we further propose that the produced fumarate may inhibit TET-mediated demethylation of a regulatory region of *mir-200ba429* in WM-266-4 cells. Its suppression will result in ZEB1 and ZEB2 (*mir-200ba429* target transcripts) upregulation and therefore initiation of EMT program (discussed in the following section). In accordance, TET3 was missing and TET2 was hardly detected (0.00) in our WM-226-4-specific proteomic profiling. Presumably, it may not be the whole melanin-positive WM-266-4 cell sub-population that carries tumorigenic potential, but rather a small group of these cells that can become oncogenic only after they acquire driver mutations in critical genetic loci (e.g. knockdown of fumarate hydratase gene or inactivation of its cognate product). Besides its presumable contribution to the rise of fumarate levels, L-tyrosine (and L-DOPA) can also induce a melanogenic program that is tightly associated with a significant upregulation of HIF-1A and its target genes [52]. Furthermore, induction of melanogenesis has been previously characterized by changes of sodium acetate and glucose metabolism [53]. Given the cardinal role of HIF-1 in cancer glucose metabolism [52, 54–56], it could be the pigmented -minor- sub-population of WM-266-4 cells that contains a stabilized (likely due to L-tyrosine-dependent signaling) HIF-1A protein (0.00) and an aberrant bioenergetic program able to provide melanotic melanoma cells with a chemotherapy (and/or radiotherapy) resistance advantage. Hence, targeting pigmented melanomas via engagement of HIF-1A specific and potent inhibitors, such as the natural flavonoid chrysin [57], may emerge as a powerful strategy for the successful therapy of the disease. Remarkably, according to several pioneer studies [7, 50, 58–60], besides its role as a major substrate and intermediate of the melanogenic pathway, L-tyrosine (and its downstream metabolic derivative L-DOPA) can also act as a hormone-like positive regulator of melanogenesis. As dictated by these findings, it must be the ability of L-tyrosine to increase the tyrosinase (TYR) activity and its melanogenic efficacy (via, for example, protein’s proper folding, enhanced exiting from the endoplasmic reticulum, carbohydrate modifications in the Golgi apparatus and transport into melanosomes) that drives the *in vivo* stimulation of melanogenesis in WM-266-4 cells (a minor sub-population). Hence, employment of specific inhibitors targeting the tyrosinase activity (e.g. *N*-phenylthiourea or D-penicillamine [48, 49]) may prove beneficial for the successful management of pigmented melanomas. Alternatively, reduction in the supply of cancer cells with L-tyrosine could compromise the melanotic phenotype of developed tumors. Given that serum components and most importantly high levels of L-tyrosine can stimulate melanogenesis in certain cellular systems [58, 59, 61–63], the DMEM growth medium that is enriched in L-tyrosine (and is also supplemented with 10% FBS) may critically control the melanogenic apparatus and melanotic character of our WM-266-4 cells. Therefore, the proteomic landscape herein described might reflect specific metabolic, signaling, oncogenic and growth adaptations of WM-266-4 cells and it could presumably differ from the one obtained under distinct experimental conditions, such as low concentrations of the L-tyrosine metabolite. Altogether, and by extending the perspective of current targeted treatments [12], the determination of both mutational and melanotic status of an advanced melanoma tumor at the time of its histological evaluation is a strategy of fundamental importance in the optimization and individualization of disease management.

Given the two different networks herein reconstructed (Fig 2A and 2B), we reason that our protein collection may derive from at least two plastically inter-converted cell sub-populations with transiently distinct phenotypes; (a) a more differentiated and non-cancer stem cell (N-CSC) one with a relatively low (as a whole sub-population) tumorigenic capacity (Fig 2A), and (b) a pluripotent cancer stem cell (CSC) one of a comparatively increased malignant potential (Fig 2B). A dynamic and plastic inter-conversion between N-CSCs and CSCs has been previously described in breast carcinoma cells with CD44 and ZEB1 critically signifying their stem-cell state [64]. Moreover, human malignant-melanoma-initiating cells (MMICs) can be defined (in certain micro-environmental settings) by the expression of ABCB5 transporter that provides them with strong self-renewal and tumorigenic advantages [65]. Intriguingly, ABCB5-expressing melanoma cells could selectively survive when exposed to the targeted BRAF^V600E^ inhibitor vemurafenib, thus indicating a role of ABCB5 in acquired chemoresistance during therapy [66]. Since all three CD44, ZEB1 and ABCB5 components can be identified in our deep-proteome catalogue, it seems that WM-266-4 may contain a CSC-like sub-population with MMIC properties that could drive cells to states of metastatic malignancy and (melanin-independent) chemotherapeutic refractoriness. A “Mascot score”-based quantification of CD44 (49.76), ZEB1 (3.68) and ABCB5 (0.00) proteins likely dictates the dynamic co-existence, with potential inter-conversion among them, of three transiently distinct sub-populations. They can genetically be characterized as CD44^+^-ZEB1^-^-ABCB5^-^, CD44^+^-ZEB1^+^-ABCB5^-^ and CD44^+^-ZEB1^+^-ABCB5^+^ with the (CSC-like) last one carrying the strongest tumorigenic capacity. Alternatively, there could grow only one CD44^+^-ZEB1^+^-ABCB5^+^ population confined in an intermediate state that is plastically directed either to a more differentiated or to a more stem-cell-like phenotype. This decision may critically depend on specific micro-environmental cues and stimuli. However, the absence of PAX5, a transcription factor whose loss in late B cells initiates lymphoma development [67], and the extremely low levels of PAX4, a family member that has been previously reported to function as tumor suppressor [68], just like they are both presented in our proteomic landscape, could provide WM-266-4 putative sub-populations with minimum de-differentiation and tumorigenic capacities. In any case, it may be the combined targeting of CD44, ZEB1 and ABCB5 oncogenic determinants that could significantly contribute to melanoma-tumor eradication and successful therapy of the disease.

### EMT/MET plasticity contributes to melanoma’s heterogeneity and chemoresistance

**Fig 5:**
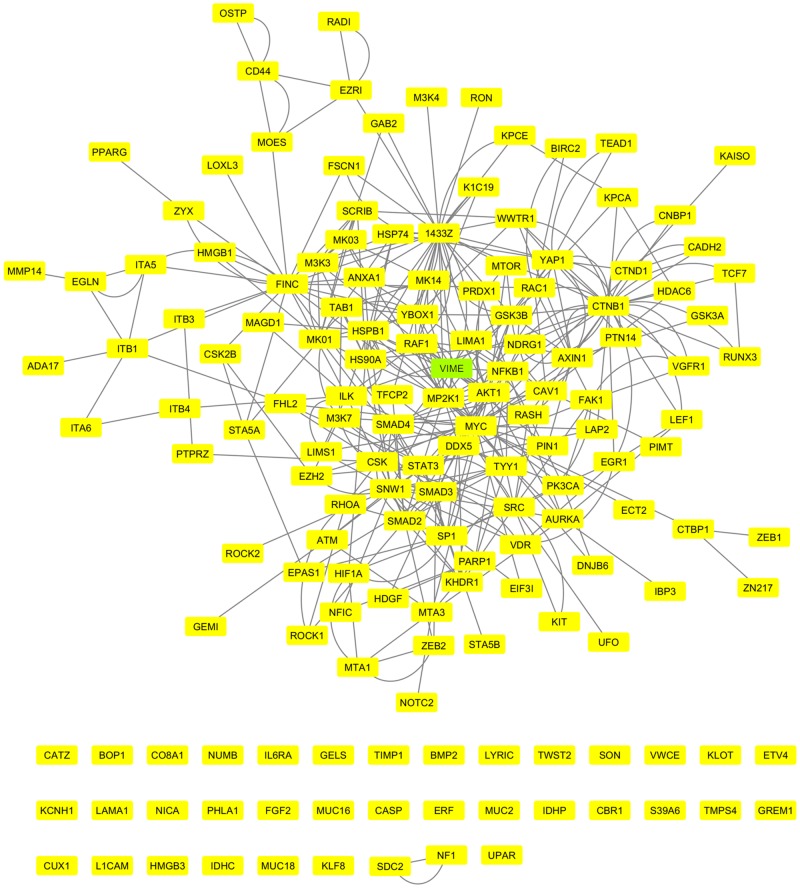
Molecular reconstruction of a WM-266-4-specific network that is tightly associated with the EMT program. The green box indicates the most decisive EMT biomarker VIM/VIME in the WM-266-4 metastatic melanoma setting. Thirty-seven EMT components could not be incorporated into the cluster. dbEMT, INTACT and CYTOSCAPE were the bioinformatics resources applied.

By applying the dbEMT bioinformatics resource, 163 unique melanoma proteins (~2.4%) out of the 6,681 total ones could be identified as EMT components (S2 Table). The remarkable accumulation of vimentin/VIM with the highest “Mascot score” (1,638.22) among all 6,681 retrieved proteins, the absence of E-cadherin/CDH1 and occludin/OCLN, and the detection of N-cadherin/CDH2 (“cadherin switch”) strongly suggest according to widely accepted criteria [69, 70] that WM-266-4 cells have undergone a typical epithelial-to-mesenchymal transition (EMT). It must be the EMT-specific transcription factors ZEB1, ZEB2 and TWIST2 [69–72], as they are recognized in our proteomic profiling, that can efficiently orchestrate metastatic melanoma cells to obtain an EMT phenotype. Through employment of (a) the INTACT bioinformatics platform that has been designed to predict molecular interactions via exploitation of their already available experimental profiles identified and (b) the CYTOSCAPE software that has been created for visualization of molecular-interaction networks, 126 (~77.3%) out of 163 EMT melanoma proteins could be organized in an integrated EMT cluster (Fig 5), thus dictating the complexity, inter-dependency, redundancy, plasticity, hierarchy and multifaceted operation of EMT components. There must be some critical -driver- interactions that have to be satisfied in order for the typical EMT program to be deployed, whereas loss of certain determinants may compromise EMT’s competence.

Surprisingly, and in contrast to the standard model [69, 70], our WM-266-4 deep-proteome catalogue proved to contain 12 keratin (KRT) type-I/II cytoskeletal family members, with KRT10 (25.56), KRT2 (14.80), KRT5 (11.87), KRT1 (8.66), KRT9 (6.53), KRT19 (5.15), KRT28 (2.72) and KRT13 (2.04) proteins being expressed at comparatively notable levels, as it is indicated by their respective “Mascot scores” (numbers in parentheses). With the exception of KRT19 that has been previously reported to characterize circulating tumor cells (CTCs) in breast cancer patients [73] and also be critically implicated in EMT and self-renewal of hepatocellular carcinoma CSCs [74], all the other KRT family members herein identified suggest that WM-266-4 cells, besides their mesenchymal character, have also gained critical epithelial features. Furthermore, the hardly detectable levels of PRRX1 (0.00), a homeobox factor whose loss is required for metastatic colonization and EMT reversion *in vivo* [75], and the enriched protein contents of NDRG1 (82.48), a potent EMT inhibitor [76–78], further corroborate that WM-266-4 melanoma cells, in addition to EMT, have also acquired a mesenchymal-to-epithelial (MET) phenotype.

Since WM-266-4 cells have originated from a metastatic skin site of human melanoma, it seems that EMT is instigated for their initial dissemination, while later on cells undergo MET upon reaching a desirable metastatic niche to seed new tumor. This composite combination of EMT and MET phenotypes herein described for the first time in a malignant melanoma cell-culture system has been recently referred to as a “metastable” state, directly reflecting the plasticity of cells to induce or reverse (MET) the EMT process [70]. The co-expression ratios of VIM and KRTs may successfully label the partial EMT or (hybrid) intermediate EMT/MET phenotypes that are likely associated with the cellular heterogeneity usually observed in solid tumors (e.g. melanomas) and their micro-environments. Hence, these non-typical EMT/MET *in vivo* gradients must be efficiently targeted for the irreversible elimination of all metastatic melanoma cell sub-populations.

**Fig 6:**
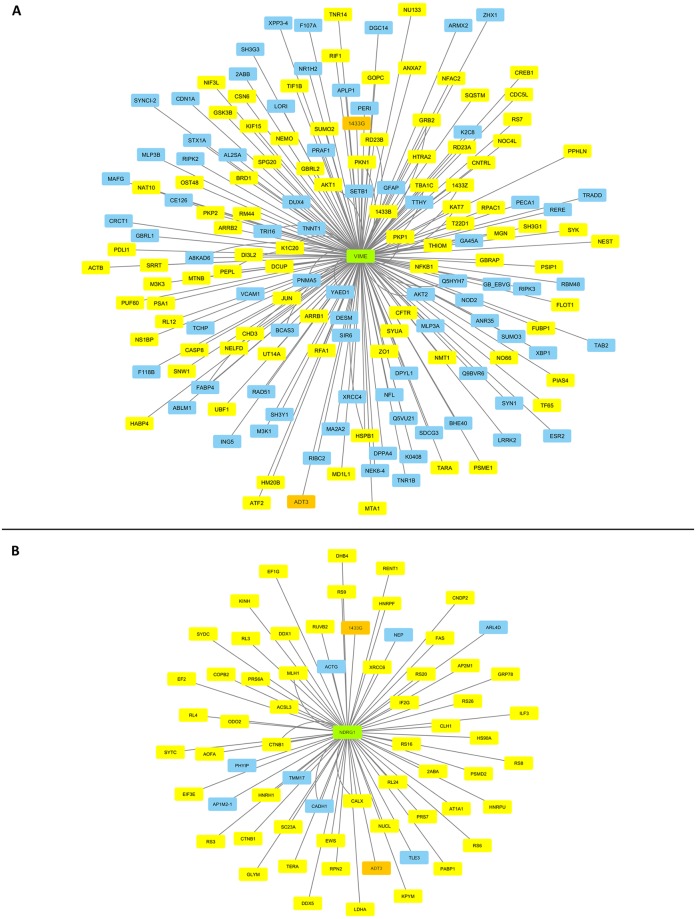
Reassembly of VIM/VIME- and NDRG1-specific interactomes in WM-266-4 melanoma cells. (A) VIM-based molecular interactome. (B) NDRG1-based molecular interactome. Green boxes indicate the EMT and MET critical determinants VIM and NDRG1, respectively. Yellow boxes denote the VIM- or NDRG1-interacting proteins identified in our WM-266-4 deep-proteome catalogue. Blue boxes point out the VIM- or NDRG1-interacting protein partners that have been missed in the present study. Orange boxes mark the two WM-266-4 proteins 1433G and ADT3 that are shared between the VIM- and NDRG1-specific interactomes. INTACT and CYTOSCAPE were the bioinformatics platforms engaged.

Considering VIM and NDRG1 as driver regulators of EMT and MET programs respectively [69, 70, 76–78], and by engaging the INTACT and CYTOSCAPE bioinformatics platforms, we reconstructed an integrated molecular-interaction network in WM-266-4 cells for each one of the two proteins examined. Both VIM (Fig 6A) and NDRG1 (Fig 6B) melanoma-specific interactomes were successfully reassembled, with NDRG1 being compared to VIM the smaller and less complicated one missing only 8 (~12.3%) out of the 65 identified interacting partners. Strikingly, even though the two networks are presented with distinct organization and composition, they have two proteins in common; the 1433G/YWHAG (41.67) and the ADT3/SLC25A6 [(mitochondrial) ADP/ATP translocase 3] (35.39). Therefore, we suggest that even though the EMT and MET programs after an initial stage of commitment operate independently from each other, they can converge on the ADT3-dependent control of cellular ATP use. It may be the ADT3 molecular switch that couples the dynamic inter-conversion between EMT and MET states with the bioenergetic demands and supplies of tumor cells. Altogether, ADT3, due to its putatively differential regulation during EMT/MET gradient acquisition, may prove as an important chemotherapeutic target for the suppression of EMT-mediated initial dissemination and MET-directed final colonization of cancer cells in human melanomas.

Providing that WM-266-4 cells have been subjected to a successive EMT and MET control, they may be composed of different sub-populations with distinct EMT/MET respective contents. For instance, and since the receptor tyrosine kinase UFO/AXL (0.00), an essential EMT effector [79], and the transcription factor PRRX1 (0.00), whose lack critically promotes MET [75], could be hardly detected in our proteomic landscape, there may exist 4 distinct but dynamically inter-converted WM-266-4 sub-populations; the AXL^+^-PRRX1^+^, AXL^+^-PRRX1^-^, AXL^-^-PRRX1^+^ and AXL^-^-PRRX1^-^ ones. Given the pivotal role of EMT in conferring chemoresistance [70, 80, 81] and more specifically the ability of activated AXL kinase to cause resistance to EGFR-targeted therapy in lung cancer [82], the putative AXL^+^-PRRX1^-^ sub-population (if selected upon chemotherapeutic pressure) could generate a melanoma clone with strong AXL^+^-mediated chemoresistance and PRRX1^-^-dependent metastatic capacity. Moreover, the clonal selection of AXL^+^ cells will likely provide melanoma tumors with an additional metastatic advantage similar to the one previously reported for breast cancer [79]. Hence, targeting AXL kinase may prove as a first-in-line class of therapy that can successfully eliminate the EMT/MET plasticity-driven melanoma-tumor heterogeneity and efficiently eradicate all metastatic melanoma cell sub-populations. Extending the currently-recruiting-patient clinical trials of the selective AXL inhibitor BGB324 in acute myeloid leukemia and non-small cell lung cancer [70, 83], a new study of BGB324 in combination with dabrafenib (an inhibitor of certain mutant-BRAF forms) in metastatic melanoma is getting ready to open for participant recruitment (https://clinicaltrials.gov) holding a promise for successful clinical management of the disease.

### Oncogenic addiction to autocrine/paracrine signaling offers new opportunities for targeted therapy of human melanoma

**Fig 7:**
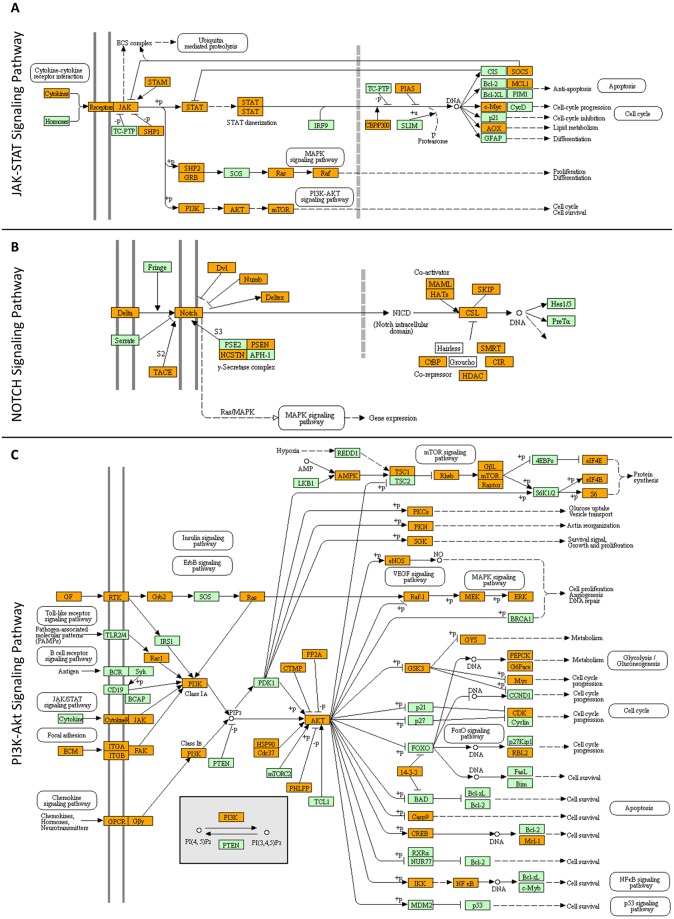
Reconstruction of molecular networks typifying: (A) “JAK-STAT signaling”, (B) “NOTCH signaling” and (C) “PI3K-Akt signaling”. Orange boxes: WM-266-4 melanoma proteins identified in the present study. Green boxes: proteins that have been missed in this study (for either technical or biological reasons). White boxes: proteins that are missing from human proteome (B). KEGG pathway maps was the bioinformatics tool employed.

Among the molecular pathways reconstructed via KEGG engagement for WM-266-4 deep proteome, only the cytokine-, DELTA-, growth factor (GF)- and OSTEOPONTIN (ECM)-dependent ones were presented with autocrine/paracrine signaling modes, respectively (Fig 7). Identification of interleukin IL17C (0.00), interleukin IL17F (0.00) and interleukin receptor IL17RD (0.00) indicates that an interleukin 17-specific (“JAK-STAT”) signaling circuit may operate in WM-266-4 cells (or minor sub-populations) critically controlling their aberrant growth and tumorigenicity (Fig 7A). Since IL17RD represents an orphan (unknown ligand) receptor [84], it could be its melanoma-specific interaction with a putative IL17C-IL17F complex that potentiates cytokine’s oncogenic-signaling capacity. Interleukin 17 induces EMT in prostate cancer [85] and lung adenocarcinoma [86], and also enhances self-renewal of glioma stem cells through an autocrine/paracrine cytokine feedback loop [87]. Hence, targeting its pathway’s components in metastatic melanoma environments will likely open a novel therapeutic window for the affected patients.

The *in silico* detection of NOTCH2 (5.33) and NOTCH4 (0.00) receptors together with DLL3 (DELTA-like protein 3) (3.50) ligand indicates the critical contribution of a “NOTCH” signaling autocrine/paracrine mechanism in WM-266-4 cell survival and growth (Fig 7B). Since NOTCH2 has been previously reported to promote proliferation and invasion in uveal melanoma [88], and also serves as a promising prognostic biomarker for oesophageal squamous cell carcinoma [89], it may play a pivotal role in maintaining derailed growth and invasiveness of melanoma cells. On the other hand, given that NOTCH4 signaling can either trigger EMT [90] or induce a MET-like phenotype [91] in certain melanoma settings, WM-266-4 cells could consist of EMT-subjected, tumor-initiating (TI; NOTCH4^+^) and MET-captured, metastatically-colonized (MC; NOTCH4^+^) sub-populations. Interestingly, DLL3 can operate as an inhibitor of NOTCH activity when both ligand and receptor are expressed in the same cell (“*in cis*”) [92–94], while DLL3 increased expression seems to essentially contribute to neuroendocrine tumorigenesis *in vivo* [95]. Providing that DLL3 when expressed “*in cis*” with NOTCH4 can suppress receptor’s signaling activity, the EMT-suffering (TI) NOTCH4^+^ (active NOTCH4; DLL3^-^) cells upon upregulation of DLL3 ligand may be transiently converted to MET-characterized NOTCH4^+^-DLL3^+^ (inactive NOTCH4) cells, rendering DLL3 a critical modulator of EMT/MET inter-conversion in metastatic melanoma. On the other hand, an “*in trans*” interaction of DLL3 of one melanoma cell (NOTCH4^+^-DLL3^+^; inactive NOTCH4), with NOTCH4 of an adjacent melanoma cell (NOTCH4^+^-DLL3^-^; active NOTCH4) could cause a receptor-signaling over-activation that promotes the clonal expansion of EMT-subjected WM-266-4 cell sub-populations. Surprisingly, the initially described developmental function of DLL3 was suggested to be the activation of NOTCH-receptor signaling [96], thus indicating its (DLL3) micro-environment-dependent dual role in cell division, growth, migration, invasion and metastasis. Altogether, the EMT/MET melanoma-specific gradients may depend on the “*in cis*” or “*in trans*” differential interactions between DLL3 and NOTCH4 proteins, while the DLL3/NOTCH2/NOTCH4 signaling axis likely emerges as a promising target for metastatic melanoma therapy. For example, DLL3^+^ melanoma cells could be specifically eliminated by a humanized anti-DLL3 monoclonal antibody conjugated to a DNA-damaging agent (e.g. a toxin), as previously reported for high-grade pulmonary neuroendocrine tumors [95]. A combination of DLL3 and BRAF^V600E^ targeted inhibitors may prove as a new regimen of high therapeutic efficacy for all stages of initiation, progression, invasion and colonization of metastatic melanoma.

WM-266-4 deep proteome was presented with low but detectable levels of the fibroblast growth factors FGF2 (0.00) and FGF13 (0.00) together with the fibroblast growth factor receptor FGFR4 (0.00), suggesting the engagement of FGF2/FGFR4 and/or FGF13/FGFR4 (“PI3K-Akt”) autocrine/paracrine signaling modes in metastatic melanoma cells (Fig 7C). Since an activated FGF2/FGFR1 autocrine loop supports proliferation and survival of uveal melanoma cells [97], and FGFR4 promotes stroma-induced EMT in colorectal cancer [98], the FGF2/FGFR4 signaling pathway may critically control melanoma initiation and progression. Moreover, upregulated expression of FGF13 seems to mediate resistance of cervical cancer cells to platinum-based drugs [99], thus rendering FGF13 an important target for single- or combination-drug therapy of malignant melanoma. Interestingly, a novel irreversible kinase inhibitor (BLU9931) that exquisitely targets FGFR4 has been successfully used for the treatment of hepatocellular carcinoma [100], while a soluble decoy receptor (GSK3052230) that sequesters FGFs and inhibits their cognate receptor-emanating signals has been recently tested in models of mesothelioma [101], hence providing a rationale for their (BLU9931 and/or GSK3052230) clinical applications (with or without vemurafenib) to therapies of metastatic melanoma patients.

In our WM-266-4 melanoma-protein collection, the identification of OSTEOPONTIN/OSTP (5.78), an extracellular matrix (ECM)-associated and chemokine-like protein [102], together with the integrin/ITG family members α_v_ (32.53), α_4_ (6.14), α_9_ (0.00), β_1_ (28.21), β_3_ (30.60) and β_5_ (32.18), whose heterodimeric complexes α_v_β_1_, α_v_β_3_, α_v_β_5_, α_4_β_1_ and α_9_β_1_ can serve as OSTP cognate receptors [102–104], indicates the operation of an OSTP/ITG autocrine/paracrine signaling system with a decisive role in human melanoma (Fig 7C). Since OSTP has proved to operate as a key regulator of EMT program [105] and strong inducer of tumor growth and angiogenesis via autocrine/paracrine mechanisms in a breast-cancer model [106], the herein presented OSTP/ITG autocrine/paracrine signaling pathway(s) must critically contribute to aberrant proliferation, enhanced angiogenesis and derailed migration of metastatic melanoma cells. Hence, each one of the two signaling components or the OSTP/ITG structural interface itself could be successfully targeted for the therapeutic management of human melanoma. For example, metastatic melanoma pre-clinical models or disease-affected patients could be treated with IPS-02001, a -novel- small-molecule protein-protein interaction (PPI) inhibitor that blocks the OSTP/ITG α_v_β_3_ structural interface [107], presumably impairing the melanoma-specific OSTP/ITG downstream signaling and thus suppressing tumor growth and expansion. However, the diversity of ITGs that can recognize OSTP dictates the probable development of resistance to the drug and further necessitates the engagement of drug cocktails and combination therapies.

### BRAF and MITF melanoma-specific interactomes converge with EMT/MET-program interface on 14-3-3/1433 family members

**Fig 8:**
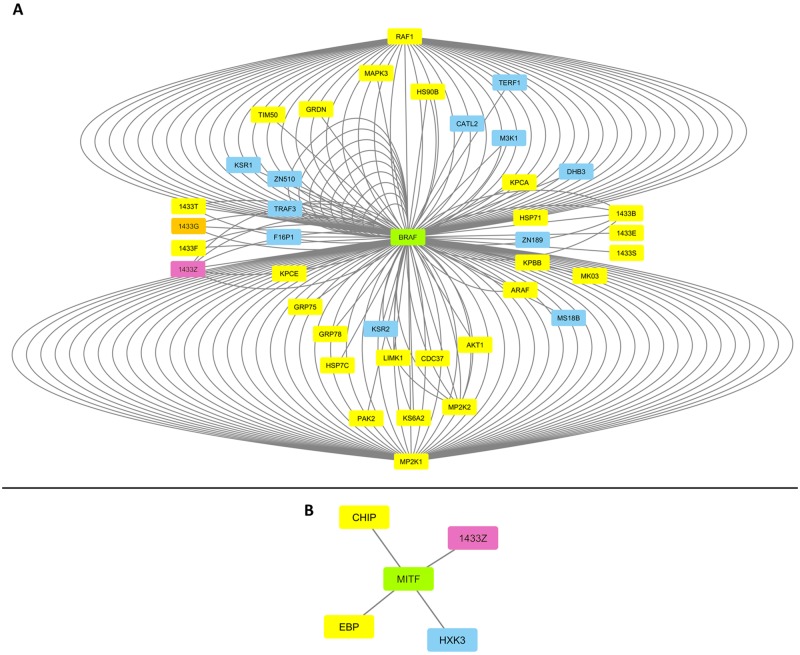
Molecular remodeling of BRAF- and MITF-specific interactomes in WM-266-4 melanoma cells. (A) BRAF-based interactome. (B) MITF-based interactome. Green boxes indicate the oncogenic determinants BRAF and MITF that are critically implicated in metastatic melanoma. Yellow boxes denote the BRAF- or MITF-interacting partners identified in our WM-266-4 proteomic landscape. Blue boxes mark the BRAF- or MITF-interacting proteins that have been missed in this study. Orange box points out the 1433G protein that is shared among the BRAF-, VIM- and NDRG1-specific interactomes in WM-266-4 melanoma cells (Fig 6). Purple boxes indicate the 1433Z family member that is shared between the BRAF- and MITF-specific interactomes in WM-266-4 melanoma cells. INTACT and CYTOSCAPE were the bioinformatics resources engaged.

Given the pivotal roles of oncogenic BRAF and MITF proteins in malignant melanoma and its therapeutic management [1, 3, 4, 6, 8, 11, 13–19, 36, 42, 108–110], and by employing the INTACT and CYTOSCAPE bioinformatics platforms, we have herein reconstructed the WM-266-4 BRAF- and MITF-specific interactomes (Fig 8). BRAF human interactome was presented with 39 partners, 28 (~71.8%) of which were identified in our WM-266-4 proteomic catalogue (Fig 8A). Surprisingly, BRAF^(V600D)^ was missing from our collection likely due to its mutation-driven change of intracellular topology that could hinder the successful extraction of the protein. As previously reported for thyroid cancer [111] and in contrast to its wild-type counterpart, mutant BRAF^V600D^ protein may be selectively localized in the mitochondria of melanoma cells not only enhancing cellular oncogenicity but also escaping drug targeting. It must be this differential attachment of BRAF^V600D^ to mitochondria that likely accounts for its absence from our proteomic landscape herein described.

Three (75%) out of the totally 4 MITF human interacting partners were recognized in WM-266-4 cells (Fig 8B). Interestingly, the BRAF- and MITF-specific interactomes proved to share the 1433Z/YWHAZ (91.73) protein, while all 7 (1433) family members (T, G, F, Z, B, E and S) were identified in our proteomic collection as BRAF interactors (Fig 8A). Among them, 1433S/SFN (12.72) was presented with the comparably lowest protein content, suggesting its tumor-suppressing role in metastatic melanoma. In agreement, loss of 1433S leads to activation of EMT [112] and promotes c-MYC-orchestrated cancer metabolic reprogramming [113], while 1433S knockdown enhances resistance of -tongue- cancer cells to chemotherapy [114]. On the other hand, as previously reported for breast cancer [115], the enriched protein levels of 1433Z may promote GLI2 (0.00) stabilization (in a minor sub-population), whose (GLI2) partnership with the -activated- SMAD2 (2.27), SMAD3 (1.80) and SMAD4 (0.00) proteins could provide melanoma cells with a strong metastatic advantage, thus rendering 1433Z a novel molecular target for cancer therapy [116] and especially malignant melanoma.

Remarkably, the BRAF/MITF and EMT/MET interactome-specific interfaces seem to converge on 1433 family members. Furthermore, the VIM, NDRG1 and BRAF respective interactomes present 1433G as their only common component (Figs 6 and 8). Providing that BRAF and MITF networks are co-regulated by 1433Z (Fig 8), while EMT and MET programs are inter-converted via 1433G control (Fig 6), it could be the 1433Z/1433G functional cross-talk and/or heterodimerization that direct the oncogenic behavior and drug response of metastatic melanoma. 1433 represents a family of dimeric α-helical, cup-shaped proteins that specifically recognize phospho-Ser/Thr sequence motifs. Since each 1433 dimer contains two adjacent phospho-peptide-binding sites, it can simultaneously interact with two ligands and serve as a versatile adaptor [117]. Hence, the interactome profiling of melanoma-specific 1433Z/1433G heterodimer must critically differ from the 1433Z/1433Z (homodimer), 1433G/1433G (homodimer), 1433Z (monomer) and 1433G (monomer) respective ones. Using combined immunoprecipitation and Western blot analysis, a strong 1433Z/1433G complex was previously detected in HEK293T cells [118], while dimerization of 1433Z proved essential for its stability and function in *Drosophila* neurons [119]. Given the pivotal role of 1433G in the prevention of centrosome amplification [120] and CEP131-dependent block of new centriolar-satellite formation [121], 1433Z may provide 1433G, in the context of a stable heterodimer, with novel activities regarding the regulation of CEP131/centrosomal protein of 131 kDa (2.38) in melanoma cells. To mechanistically associate centrosomal homeostasis with EMT/MET-program inter-conversion is an interesting issue that needs to be further explored. Targeting the 1433Z/1433G heterodimer may critically modulate centrosome amplification, aneuploidy proneness and metastatic aggressiveness of melanoma tumors. Altogether, it seems that destabilization of 1433Z/1433G complex may offer novel therapeutic perspectives to malignant melanoma. To this direction, the RB-011 and RB-012 compounds that disrupt 1433 dimers at low micromolar concentrations and compel -lung- cancer cells to apoptosis [122] could be successfully exploited (with or without vemurafenib) as a novel approach to treat metastatic melanoma. A new era of PPI inhibitors has recently emerged, through high-scale screens of chemical libraries and advanced computational modeling [123, 124], for several oncogenic targets including the 1433 family members and their dimeric complexes.

### Metastatic melanoma cells are addicted to metabolic pathways controlling ammonia, propanoate and sulfur homeostasis

**Fig 9:**
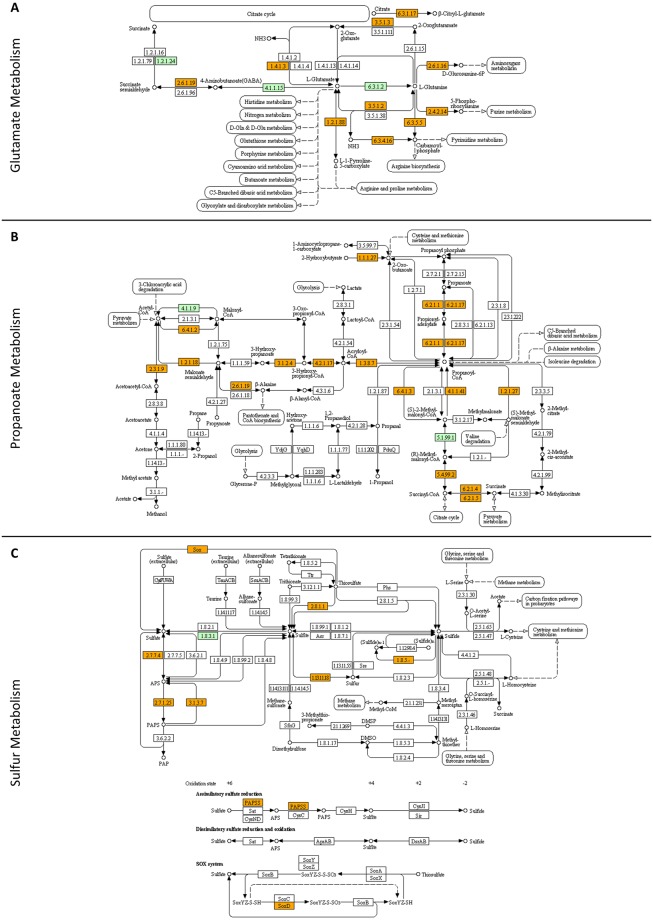
Reconstruction of molecular networks that regulate: (A) “glutamate metabolism”, (B) “propanoate metabolism” and (C) “sulfur metabolism”. Orange boxes: WM-266-4 melanoma proteins that have been identified in the present study. Green boxes: proteins that have been missed in this study (for either technical or biological reasons). White boxes: proteins that are missing from human proteome. The bioinformatics platform employed was the KEGG pathway maps.

Engagement of KEGG bioinformatics platform allowed the successful reconstruction of “glutamate”-, “propanoate”- and “sulfur”-specific metabolic networks in WM-266-4 melanoma cells (Fig 9). It has been recently reported that the metabolic conversion of “glutamate” to alpha-ketoglutarate is mediated in quiescent cells by glutamate dehydrogenase (GLUD) and in proliferating cells by transaminase (TA) activities [125]. Thus, the identification of GLUD1 (62.56) and GLUD2 (28.00) proteins, together with PSAT1 (27.44), GOT1 (22.73) and GOT2 (31.62) TA family members (with rather comparable expression levels among them), indicates the co-existence of WM-266-4 distinct sub-populations that carry different proliferation capacities (comparatively low or high) and “glutamate” metabolic profiles (GLUD- or TA-dependent), thus underscoring the oncogenic addiction of metastatic melanoma to “glutamate” metabolism (Fig 9A). GLUD-mediated catabolism of “glutamate” results in production of ammonia (NH_3_/NH_4_^+^: pH-dependent inter-converted molecular moieties [126]) [125, 127] that if is constantly accumulated and not secreted or detoxified can kill the cell. Since most tumors contain large numbers of non-proliferating malignant cells [127], in order for them to survive, a paracrine mechanism of ammonia-homeostasis maintenance may have evolved. Based on the ability of ammonia to induce autophagy [128–131] and the addiction of oncogenic Ras-driven tumors to basal autophagy [132, 133], it is possible that in a given tumor of metastatic melanoma the non- or low-proliferating cells, through the GLUD-directed pathway of “glutamate” catabolism, can release ammonia in their micro-environment, which next flows into the adjacent high-proliferating (positive for BRAF^V600E^) cells strongly inducing, via paracrine signaling, basal autophagy and hence promoting tumor survival and growth. In accordance, the low levels of CPS1 (0.00), a critical regulator in urea synthesis that uses NH_3_ to produce carbamoyl phosphate [134, 135], in our proteomic catalogue further indicate the important role of NH_3_ signaling in WM-266-4 oncogenicity. Targeting the ammonia-triggered autophagic machinery and/or the NH_3_/NH_4_^+^ membrane transporters [126] may prove as a powerful strategy for successful therapy of malignant melanoma. Alternatively, if ammonia is not adequately secreted, it can destroy the non- or low-proliferating melanoma cells, which, similarly to a previous report [136], during their dying process may be able to recruit and activate neutrophils in an HMGB1-dependent manner. Activated neutrophils likely produce pro-inflammatory cytokines (e.g. TNF) that stimulate angiogenesis and promote migration of high-proliferating melanoma cells towards the new blood vessels, thus facilitating cancer’s spread to distant organs. Inactivating the HMGB1 protein (201.31) via treatment of malignant melanoma cells with inflachromene, a targeted blocker of HMGB1/HMGB2 cytoplasmic localization and extracellular release [137], could significantly restrict metastatic expansion of the disease.

The successful reassembly of “propanoate” molecular network in WM-266-4 cells (Fig 9B) likely indicates the metabolic addiction of metastatic melanoma to propanoyl-CoA/propionyl-CoA intermediate biosynthetic product. 2-methyl-citrate can be generated in bacteria [138–140] by the 2-methyl-citrate synthase (EC 2.3.3.5; Fig 9B) that uses propionyl-CoA as one of its substrates [139, 140]. Therefore, in the case of infections frequently being developed due to chemotherapy-induced immunodeficiency, tumors of melanoma-affected patients may produce high levels of propionyl-CoA that can be successfully utilized by surrounding bacteria to synthesize 2-methyl-citrate. Since 2-methyl-citric acid has been recently reported to inhibit GLUD activity [141], the GLUD-dependent non- or low-proliferating cells will be likely eliminated, thus changing the balance of non-/low- and high-proliferating cells in a melanoma tumor. Whether this promotes or reduces oncogenicity can be demonstrated by the engagement of targeted inhibitors against “propanoate”-metabolism determinants. Hence, in mice that carry melanoma tumors and are also infected with *Mycobacterium tuberculosis*, the administration of V-13-009920, a targeted inhibitor of *M*. *tuberculosis* 2-methyl-citrate synthase [139], will clarify the either beneficial or detrimental role of the bacterially derived 2-methyl-citrate metabolite in metastatic melanoma.

The almost completely reconstructed network of “sulfur” metabolism in our WM-266-4 deep-proteome profiling (Fig 9C) suggests its indispensable contribution to melanoma growth and progression. In agreement, the ^32^S stable-isotope enrichment of “sulfur” in the blood of hepatocellular carcinoma patients has been previously proposed to serve as a new biomarker for the disease, as it seems to partly originate from tumor-derived sulfides [142]. Moreover, hydrogen sulfide (H_2_S) proved able (a) to maintain colon cancer cells’ bioenergetic capacity, thus supporting tumor survival and proliferation, and (b) to promote angiogenesis, thereby providing tumor with nutrients and factors required for cell migration, invasion and metastasis [143]. Successful targeting of the H_2_S-producing enzyme cystathionine-β-synthase/CBS (0.00), with use of its specific inhibitor amino-oxy-acetic acid [143], in metastatic melanoma will likely emerge as a novel and promising therapeutic approach for the disease.

## Conclusions

Our mass-spectrometry-based draft map of WM-266-4 metastatic-melanoma proteome embraces the hitherto largest single cell-line-derived protein collection of the disease. However, given the melanotic character of WM-266-4 cells and the ability of melanin to compromise the LC-MS/MS performance [144], the proteomic content of melanin-positive sub-population may be reduced as compared to the melanin-negative one. Nevertheless, our WM-266-4 deep proteome will likely complement the available human genome and transcriptome information to accelerate basic and translational research in the years to come, through an in-depth analysis and better comprehension of the gene-protein-pathway-network workflow in melanoma initiation and progression. Moreover, it can be successfully integrated in the “core cancer proteome (CCP)”, as this has been originally initiated by carrying out a global proteome analysis of the NCI-60 cell-line panel [145]. Of note is the observed increase of more than 35% in the number of WM-266-4 unique proteins (this study) as compared to each one of the NCI-60 cell lines previously examined [145]. Our WM-266-4 deep-proteome database enables a wide range of melanoma-reference analyses, including among others the: (a) expression profiles for proteins of interest, (b) “multifeature/panel biomarkers” for malignancy grade and (c) complex molecular signatures for drug sensitivity or resistance. Comparison of our WM-266-4 proteomic landscape with the available draft map of human proteome [146–148] may open a new mechanistic window in the understanding of processes controlling cellular transformation and malignancy commencement. Similarly, and by expanding a previous report [149], a comparative and high-scale proteomics approach between the primary WM-115 and metastatic WM-266-4 matched melanoma cell lines (manuscript in preparation) could allow the identification of key proteins that are able to orchestrate the progression and metastasis of human melanoma. Commensurate advances in the core technology of mass spectrometry, computational proteomics and bioinformatics will provide unprecedented insights into the composition, structure, function and regulation of human oncoproteomes. Furthermore, they will constitute an excellent starting point for modeling cancer cells in order for the biomedical society to design new regimens that can successfully fight off chemoresistance frequently being developed during therapy.

## Supporting information

S1 Table Protein library (n = 6,681 deep-proteome components) of WM-266-4 human metastatic melanoma cells (Microsoft Excel format file), indicating for each identified member its: (a) (UNIPROT) “accession” (number), (b) (name) “description” and (c-h) fundamental features of the nLC-MS/MS proteomics analysis employed, including among others the: (c) (Mascot) “score”, (d) (sequence) “coverage”, (e) (number of) “unique peptides” (N = 23,191 tryptic fragments), (f) (number of) “AAs” (amino acid residues), (g) “MW” (molecular weight) in kDa and (h) (calculated) “pI” (isoelectric point).
(XLSX)

S2 Table Deep-proteome catalogue (163 unique members) of WM-266-4 human metastatic melanoma proteins (Microsoft Excel format file) that have been classified (through application of the dbEMT bioinformatics resource) as *bona fide* EMT-program components. “Accession” (UNIPROT number) and “description” (name) of each identified protein, together with cardinal characteristics of the nLC-MS/MS proteomics analysis employed, including among others the (Mascot) “score”, (sequence) “coverage”, (number of) “unique peptides”, (number of) “AAs” (amino acid residues), “MW” (molecular weight) in kDa and (calculated) “pI” (isoelectric point), are shown.
(XLSX)
